# Cognitive parameters can predict change of walking performance in advanced Parkinson’s disease – Chances and limits of early rehabilitation

**DOI:** 10.3389/fnagi.2022.1070093

**Published:** 2022-12-22

**Authors:** Johanna Geritz, Julius Welzel, Clint Hansen, Corina Maetzler, Markus A. Hobert, Morad Elshehabi, Henrike Knacke, Milda Aleknonytė-Resch, Jennifer Kudelka, Nico Bunzeck, Walter Maetzler

**Affiliations:** ^1^Department of Neurology, University Hospital Schleswig-Holstein, Kiel, Germany; ^2^Department of Psychology, University of Lübeck, Lübeck, Germany; ^3^Center of Brain, Behavior and Metabolism (CBBM), University of Lübeck, Lübeck, Germany

**Keywords:** Parkinson’s disease, geriatric care, cognition, straight walking, dual task, wearable sensors, fear of falling, depression

## Abstract

**Introduction:**

Links between cognition and walking performance in patients with Parkinson’s disease (PD), which both decline with disease progression, are well known. There is lack of knowledge regarding the predictive value of cognition for changes in walking performance after individualized therapy. The aim of this study is to identify relevant predictive cognitive and affective parameters, measurable in daily clinical routines, for change in quantitative walking performance after early geriatric rehabilitation.

**Methods:**

Forty-seven acutely hospitalized patients with advanced PD were assessed at baseline (T1) and at the end (T2) of a 2-week early rehabilitative geriatric complex treatment (ERGCT). Global cognitive performance (Montreal Cognitive Assessment, MoCA), EF and divided attention (Trail Making Test B minus A, delta TMT), depressive symptoms, and fear of falling were assessed at T1. Change in walking performance was determined by the difference in quantitative walking parameters extracted from a sensor-based movement analysis over 20 m straight walking in single (ST, fast and normal pace) and dual task (DT, with secondary cognitive, respectively, motor task) conditions between T1 and T2. Bayesian regression (using Bayes Factor BF_10_) and multiple linear regression models were used to determine the association of non-motor characteristics for change in walking performance.

**Results:**

Under ST, there was moderate evidence (BF_10_ = 7.8, respectively, BF_10_ = 4.4) that lower performance in the ∆TMT at baseline is associated with lower reduction of step time asymmetry after treatment (*R*^2^_adj_ = 0.26, *p* ≤ 0.008, respectively, *R*^2^_adj_ = 0.18, *p* ≤ 0.009). Under DT walking-cognitive, there was strong evidence (BF_10_ = 29.9, respectively, BF_10_ = 27.9) that lower performance in the ∆TMT is associated with more reduced stride time and double limb support (*R*^2^_adj_ = 0.62, *p* ≤ 0.002, respectively, *R*^2^_adj_ = 0.51, *p* ≤ 0.009). There was moderate evidence (BF_10_ = 5.1) that a higher MoCA total score was associated with increased gait speed after treatment (*R*^2^_adj_ = 0.30, *p* ≤ 0.02).

**Discussion:**

Our results indicate that the effect of ERGT on change in walking performance is limited for patients with deficits in EF and divided attention. However, these patients also seem to walk more cautiously after treatment in walking situations with additional cognitive demand. Therefore, future development of individualized treatment algorithms is required, which address individual needs of these vulnerable patients.

## 1. Introduction

People with advanced Parkinson’s disease (PD), one of the most common age-associated neurodegenerative disorders, are particularly affected by a deterioration of their walking performance as well as cognitive deficits, depression, and anxiety (so-called non-motor symptoms (Yogev-Seligmann et al., 2008; Avanzino et al., 2018). These are associated with reduced mobility, increased need for assistive devices (e.g., walking aids), increased risk of falls, injuries, and acute medical care as well as the probability of institutionalization as well as treatment intervention outcomes (Rochester et al., 2004; Mirelman et al., 2019; Vila et al., 2021; Zanardi et al., 2021), and can all significantly impact independent living and quality of life (Snijders et al., 2007; Hobson and Meara, 2015; Haertner et al., 2018; Nonnekes et al., 2018; Rutten et al., 2021). However, the role of cognitive and affective non-motor symptoms in the change of walking performance after early individualized rehabilitation in acutely hospitalized patients with advanced PD is still not sufficiently understood.

With the help of modern sensor technology, some progress has been made in the last 20 years regarding the identification and classification of quantifiable disease-specific walking profiles of PD as well as their progression, for instance on the basis of spatio-temporal parameters measured with inertial measurement units (IMUs, Maetzler et al., 2016; Schlachetzki et al., 2017; Del Din et al., 2019; Bouça-Machado et al., 2020; Alberto et al., 2021; Bouça-Machado et al., 2021). Individuals with PD differ from healthy individuals in several domains of walking, meaning that they tend to walk more slowly and asymmetrically (e.g., with higher step time asymmetry) with delayed rhythm (e.g., with higher step and stride time as well as higher walking variability (e.g., higher step time, higher double limb support variability (Mirelman et al., 2019; Bouça-Machado et al., 2020; Vila et al., 2021; Zanardi et al., 2021). However, as recently reviewed, evidence was inconsistent or lacking for several parameters in terms of both validity and responsiveness, while others (e.g., gait speed, step time, stance time, double limb support or asymmetry measures) showed consistent evidence in different disease conditions (Polhemus et al., 2021). Although these studies have contributed to a better understanding of impaired walking performance in PD in recent years, heterogeneity in the selection of parameters is evident. Also, the clinical utility of individual parameters, especially for the treatment of patients with advanced PD, has not been sufficiently investigated (Mirelman et al., 2019; Bouça-Machado et al., 2020; Polhemus et al., 2021).

Associations of non-motor symptoms such as impaired cognitive performance, especially deficits in divided attention and executive functions (EF, so-called fronto-striatal associated functions of cognitive flexibility, set shifting, and working memory (Owen, 2004) and dementia with reduced walking performance, higher fall risk, and reduced quality of life in individuals with PD have also been found in several studies (reviewed in (Owen, 2004; Koerts et al., 2009, 2011; Dirnberger and Jahanshahi, 2013; Domingos et al., 2015; Lauretani et al., 2016; Moustafa et al., 2016; Avanzino et al., 2018). Recent studies in people with PD with and without mild cognitive impairment (MCI) have shown that deficits in EF are associated with reduced gait speed, lower stride length, and increased gait variability, especially under DT conditions (Yogev et al., 2005; Rochester et al., 2008; Yogev-Seligmann et al., 2008; Lord et al., 2010, 2011; Plotnik et al., 2011; Amboni et al., 2012; Smulders et al., 2013; Wild et al., 2013; Rochester et al., 2014; Stegemöller et al., 2014; Varalta et al., 2015; Maidan et al., 2016; Hobert et al., 2017; Nieuwhof et al., 2017; Salazar et al., 2017; Salkovic et al., 2017; Mirelman et al., 2018; Hillel et al., 2019; Johansson et al., 2021; Liguori et al., 2021; Amboni et al., 2022). Meanwhile, higher global cognitive performance (measured with the Mini Mental State Examination) was found to be determinant for maintenance of positive long-term effects of home-based physical training in individuals with advanced PD (Nieuwboer et al., 2002). Also, depression and FOF are associated with increased walking variability as well as decreased walking speed and quality of life in individuals with PD (Rochester et al., 2008; Brozova et al., 2009; Jacob et al., 2010; Rahman et al., 2011; Allen et al., 2013; Lord et al., 2013; Lindholm et al., 2014; Albay and Tutuncu, 2021; Atrsaei et al., 2021; Dragašević-Mišković et al., 2021). FOF is also considered a determinant of increased risk of falling (Allen et al., 2013) alongside with avoidance of physical activity in situations with increased fall-related activity (Kader et al., 2016). However, most of these studies related to cognitive and affective non-motor symptoms and their association with walking performance in individuals with PD do not consider the non-motor symptoms as predictive characteristics for post interventional change in walking performance. In addition, in the advanced disease stage (i.e., severe motor symptoms or motor fluctuations), the use of a walking aid as well as having mild cognitive impairment (MCI) or dementia are often considered as exclusion criteria.

Over the past 20 years, a substantial amount of research has been conducted on both pharmacological and non-pharmacological treatment options for walking impairments in PD (Smulders et al., 2016; Debû et al., 2018; Ni et al., 2018; Bouça-Machado et al., 2020; Radder et al., 2020). Especially in the later stages of PD, as pharmacological treatment effects become increasingly insufficient, rehabilitation and physical training programs have been identified to have a crucial complementary role in improving motor symptoms, including impaired walking (reviewed in (Bloem et al., 2015; Dietrichs and Odin, 2017; Domingos et al., 2018). Since DT walking situations can be daily-relevant for individuals with PD, DT interventions have been evaluated over the past years- with promising results mainly from a multi-centered randomized controlled trial with 120 patients with PD (Strouwen et al., 2017; Geroin et al., 2018; Strouwen et al., 2019). However, while gait speed was identified as consistently responsive (which is the most commonly used walking parameter in treatment efficacy studies focusing on walking impairment), there is still a gap of knowledge with regard to responsiveness to treatment of various spatio-temporal walking parameters (Polhemus et al., 2021; Scherbaum et al., 2022). Hence, a “one-size-fits-all” treatment approach may not mirror the complexity of the disease adequately (Ginis et al., 2017; Witt et al., 2017; Nonnekes and Nieuwboer, 2018; Serrao et al., 2019). Therefore, individualized, skilled (i.e., goal-driven), multimodal therapy approaches as established as common practice in geriatric rehabilitation are receiving more and more attention as potential treatment models for older patients with advanced PD (Nonnekes and Nieuwboer, 2018; Swanson and Robinson, 2020). However, in order to plan and implement individualized walking rehabilitation in an evidence-based manner, practitioners need to know which characteristics of the patient and their condition predict the efficacy of such training approaches, intensities, and durations.

The study presented here aimed to identify relevant cognitive and affective characteristics (measured with a comprehensive geriatric assessment, CGA) in advanced PD at admission to an acute inpatient stay at a neurogeriatric ward that are associated with changes in spatio-temporal walking parameters after 2 weeks of early rehabilitative geriatric complex treatment (ERGCT). We hypothesize that preexisting cognitive impairment, especially deficits in EF and divided attention, together with presence of depressive symptoms and FOF have a constraining effect on change in walking performance in terms of improvement during early rehabilitation.

## 2. Materials and methods

Data for this study were collected as part of the multicenter, exploratory, observational study “Cognitive and Motor interactions in the Older population” (ComOn). The ComOn study examines the association of cognitive, motor, and clinical characteristics (measured with quantitative and digital parameters of an extended CGA) in acutely hospitalized geriatric patients (at least 50 years old and have at least one chronic disease) during a stay on a geriatric ward of a Neurological Department of a University Hospital in Germany. The main aims of the study are to gain a better understanding of complex interactions of multifaceted geriatric symptoms and evaluate the efficacy of individualized geriatric inpatient treatment. Detailed information on all examinations performed have been published in the study protocol (Geritz et al., 2020).

The analyses presented here focus on the prognostic value of non-motor parameters of a CGA in patients with advanced PD on acute inpatient admission for the change in walking performance under ST and DT after 2 weeks early rehabilitative geriatric complex treatment (ERGCT, German Version of Operation and Procedure Code for hospitals (OPS) number 8–550.1). Data were collected between October 2017 and August 2021 at the Department of Neurology, University Hospital Schleswig-Holstein Campus Kiel (Germany). A written informed consent was obtained from all patients and, if applicable, their legal representatives (e.g., due to cognitive impairment or dementia). The study was reviewed by the ethics committee of the Medical Faculty of the University of Kiel (Ethics application number D 427/17).

### 2.1. Participants

For these analyses, data of N = 47 patients with PD diagnosed according to the Movement Disorder Society (MDS) clinical diagnostic criteria for PD were included (Postuma et al., 2015; Marsili et al., 2018). Participants were included if (i) they fulfilled the inclusion criteria of the ComOn study, i.e., were 50 years or older, able to walk independently over at least 3 m with or without walking aid, and had sufficient hearing and visual acuity as well as sufficient speech comprehension as judged by the investigator and (ii) received 2 weeks of ERGCT on the neurogeriatric ward. Patients were mainly administered to the inpatient stay for reasons of deterioration in mobility or walking ability, general condition, and actual falls or reduced drug effects. Patients with previously described mild cognitive impairment (MCI) or mild to moderate dementia in their medical record as well as patients with severe motor symptoms measured with the MDS-revised version of the motor part of the Unified Parkinson’s Disease Rating Scale (MDS-UPDRS III, (Goetz et al., 2008) were included. Patients were excluded if they were suffering from delirium or other severe disorders of consciousness (clinical diagnosis), were below the cut-off of ≤5 points for severe dementia in PD in the Montreal Cognitive Assessment (MoCA, (Nasreddine et al., 2005; Lawton et al., 2016), or had more than two falls in the past week due to safety reasons in the motor assessment.

### 2.2. Early rehabilitative geriatric complex treatment

ERGCT was performed in the neurogeriatric ward by a multi-professional geriatric team according to the guidelines and recommendations of the German geriatric societies (Meier-Baumgartner et al., 1998; Bundesverband Geriatrie, 2021). This included at least 14 days of skilled treatment with at least one daily session of clinical therapy by trained therapists (30 min per session) and with at least two disciplines involved (occupational, physical, and/or speech therapy). Contents of the therapy sessions were set according to an individualized indicated treatment plan. General core aspects of the treatment included strength, endurance, and balance training, combined cognitive-motor training as well as training in activities of daily living. Clinical therapists were not involved in assessment nor analyses and interpretation of the collected data, and the individualized treatment plans were not adapted for study purposes. For the analyses presented here focusing on walking performance, only the number of sessions for physical and occupational therapy was considered.

### 2.3. Procedure

Patients were assessed within 2 days after admission (T1) to and before discharge (T2) from the neurogeriatric ward. At T1, a detailed medical history as well as self-reporting questionnaires on various behavioral and clinical aspects were taken. An extended CGA was carried out (see 2.4.2) to assess non-motor symptoms, followed by a comprehensive movement analysis using inertial measurement units (IMUs, see also 2.4.4) on a designated area on the ward corridor (>3 m broad, well-lit). Each of these two latter assessments took about 60 to 90 min with a break of at least 60 min in between. Cognitive parameters were assessed during a neuropsychological assessment. Questionnaires were handed out to the patients during the admission interview with the request to complete them independently by the next day. Patients were offered help when needed. At T2, the movement analysis was carried out, preferably, at a similar daytime as T1. All patients were examined in medication ON state. For this purpose, medication was administered in close consultation with the clinic staff and the medication schedule was taken into account in order to provide a suitable time interval before the measurement.

### 2.4. Measures

#### 2.4.1. Demographical and clinical parameters

Demographical characteristics like age, gender, years of education, and geriatric aspects (e.g., care level, frailty, actual pain, problems with vision and hearing, and urinary incontinence) were collected during the medical history interview. The geriatric screenings used for this purpose are described in more detail in the ComOn study protocol (Lachs et al., 1990; Bellmann et al., 2013; Geritz et al., 2020). Clinical aspects such as PD duration, previously described cognitive deficits or dementia as well as the number of occupational and physical therapy sessions between T1 and T2 were extracted from medical record. The levodopa equivalent daily dose (LEDD, Tomlinson et al., 2010) was determined based on the medication schedule at admission.

The severity of motor-symptoms was measured using the MDS-UPDRS III (Goetz et al., 2008) by defining scores as ≤30 (mild), 30 to 60 (moderate), and > 60 (severe) for PD motor state (adapted from (Martínez-Martín et al., 2015). Furthermore, using the three related Items of the MDS-UPDRS III, the occurrence of dyskinesia (i.e., involuntary, random movements) during the examination as well as their impact on the rating of the MDS-UPDRS III, the occurrence of freezing of gait (FOG), and the modified Hoehn and Yahr Scale were recorded (Goetz et al., 2007, 2008, 2014).

#### 2.4.2. Comprehensive geriatric assessment of non-motor symptoms

##### 2.4.2.1. Global cognitive performance and executive function

Global cognitive performance was measured using the MoCA (Nasreddine et al., 2005). The total score ranges from 0 to 30 points, one extra point is given for 12 or less years of education. Cut-offs are at <26 points for MCI (sensitivity of 90%, specificity of 75%) and < 21 points for dementia (sensitivity of 81%, specificity of 95%) in patients with PD (Dalrymple-Alford et al., 2010).

EF and divided attention were measured using the paper-pencil speed test Trail Making Test (TMT, Reitan and Wolfson, 1985). The TMT consists of two parts, TMT A and TMT B (Strauss et al., 2006). Components of perceptual tracking as well as processing speed are captured by both tasks. More complex EF such as set shifting and alternating sequencing (subdomains of cognitive flexibility) and divided attention are captured additionally by part B (Strauss et al., 2006; Lamberty and Axelrod, 2012; Lezak et al., 2012). The recommended difference index ΔTMT (processing time in seconds (s) of TMT B minus TMT A) was calculated (Lamberty et al., 1994; Axelrod et al., 2000; Hester et al., 2005; Vazzana et al., 2010; Hobert et al., 2011; Lamberty and Axelrod, 2012). As this derived score corrects for processing speed, it therefore provides a better index of EF and was used for the analyses presented here.

##### 2.4.2.2. Depressive symptoms

Depressive symptoms within the last 14 days were assessed using the screening questionnaire for geriatric patients *Depression im Alter Skala* (DIA-S, Heidenblut and Zank, 2010). The DIA-S consists ten dichotomous items (scoring “0” and “1”). The total sum score ranges from zero to ten points and cut-offs range from ≤2 points (no depressive symptoms), 3 points (depression suspected) to ≥4 points (clinically relevant depression is likely). The DIA-S shows good reliability (Cronbach’s alpha = 0.84) and convergent validity as well as high sensitivity (0.81 to 0.92) in geriatric patients and is straightforward to use in the clinical setting (Heidenblut and Zank, 2014; Wunner et al., 2022).

##### 2.4.2.3. Fear of falling

FOF was assessed using the international version of the Falls Efficacy Scale (FES-I, Yardley et al., 2005; Delbaere et al., 2010). The FES-I captures concerns about falling in specific daily activities with 16 items in a four-point response format (0 = “not at all concerned” to 4 = “very concerned”). The total score is between 16 and 64 points with a cut-off of ≥23 points for high concern to fall (Delbaere et al., 2010). The FES-I shows good reliability (Cronbach’s alpha = 0.79) as well as convergent and predictive validity with regard to physical and psychological aspects (Delbaere et al., 2010).

#### 2.4.3. Straight walking performance

##### 2.4.3.1. Walking conditions

From the comprehensive movement analysis, data for straight walking performance at T1 and T2 during four walks of a marked straight distance of 20 m under ST and DT walking conditions were considered for this study. For each walk, a different condition was set with increasing task complexity. Patients were allowed to use a walking aid, if needed. If patients had the capacity to perform all four walking conditions, the assessment was conducted in the following order: First condition *ST fast pace* (covering the distance walking as fast as possible without running), second condition *ST normal pace* (walking at a self-selected comfortable speed), third condition *DT walking-motor* (checking predetermined boxes with a cross as quickly as possible with a pen on a clipboard while walking at fast pace), and fourth condition *DT walking-cognitive* (consecutively subtracting seven from a given three-digit number as fast as possible while walking at fast pace). The third condition was only possible for patients who did not require a walking aid as the checking boxes task while walking required the use of both arms. For the fourth condition, the given three-digit number was altered between T1 and T2 to avoid possible learning effects. If patients were unable to complete all walking conditions, the less complex tasks were prioritized.

##### 2.4.3.2. IMU system

The CE-certificated IMU-system RehaGait® (Hasomed, Magdeburg, Germany (Byrnes et al., 2018) was used. For these analyses, data were collected from the IMU attached with velcro-straps to the patient’s lower back at the level of the fifth lumbar vertebra. The IMU includes a triaxial accelerometer (±16 g) and a triaxial gyroscope (±2000/s). Collected data (sampling frequency of 100 Hertz) were transmitted simultaneously *via* Bluetooth to a tablet with the RehaGait® application modified for the ComOn study in cooperation with the manufacturer.

##### 2.4.3.3. Extraction of walking parameters and calculation of change in walking performance

IMU raw data were analyzed using a validated algorithm for step detection in PD to calculate the ten spatio-temporal walking parameters total number of steps, gait speed (distance divided by measurement duration, m/s), mean step time (s), mean stride time (s), mean swing time (s), mean stance time (s), mean double limb support time (DLS, s), mean double limb support time variability (DLSV, s; square rooted sum of variance of DLS for each foot divided by two), mean step time asymmetry (ASYM, s; absolute difference between mean step time difference between both feet), and step time variability (STV, s; square rooted sum of variance of step time for each foot divided by two) (Pham et al., 2017). For all walking parameters (except for gait speed), minimal detectable change (MDC) was examined in neurogeriatric subsample of the ComOn study for ST normal pace as described in detail in (Hansen et al., 2022). A linear correction of all parameters (except number of steps and gait speed) was applied to normalize for gait speed (to 1 m/s), as recommended in previous biomechanical studies on sensor-based walking parameters (Warmerdam et al., 2021).

The change in walking performance after ERGCT was calculated for each of the extracted and corrected walking parameter as the difference (Δ, delta) between T1 and T2:


$$\Delta \mathrm{walking parameter}={\mathrm{paramter}}_{\mathrm{T2}}-{\mathrm{parameter}}_{\mathrm{T1}}.$$

The evaluation of the direction of this difference depends on the respective parameter and the overall profile. Here, a positive value for Δgait speed corresponds to an increased gait speed (i.e., patients walk faster at T2 than at T1, which means an improvement), while a negative value for ΔASYM corresponds to decreased ASYM (i.e., the gait pattern of the patients is more symmetric at T2 than at T1, which means an improvement), values around zero indicate no change between the two points of measurements.

### 2.5. Statistics

To address the scientific question which cognitive and affective non-motor symptoms in patients with advanced PD may have predictive value for the change in straight walking performance after 2 weeks individualized treatment, both Bayesian regression models and multiple linear regression models were calculated for all four walking conditions. Deltas of the walking parameters were set as dependent variables (Δnumber of steps, Δgait speed, Δstep time, Δstride time, Δswing time, Δstance time, ΔDLS, ΔDLSV, ΔASYM, and ΔSTV). Each model included the MoCA total score or ΔTMT as well as DIA-S and FES-I total scores as predictors and MDS-UPDRS III total score, use of a walking aid (except for DT walking-motor), and age and gender as covariates. Regression models were calculated separately for the two predictors MoCA and ΔTMT in order to avoid multicollinearity as well as for an exploratory differentiation between global cognitive performance and EF. For patients with one missing single item in the DIA-S (n = 2, rate of completeness 90%) or a maximum of two missing single items in the FES-I (n = 7, rate of completeness of 88%), the missing values were imputed using the individual median (for DIA-S) or mean imputation (for FES-I, Shrive et al., 2006). The total scores were subsequently recalculated for these patients. The Bayes factor BF_10_ was estimated (using the Bayesian Information Criterion, BIC, (Glen, 2018) as a measure for strength of evidence between two different scientific theories (H0 vs. H1) provided by the data (H1 here: cognitive and affective non-motor symptoms can predict change in walking performance (Kass and Raftery, 1995; Wagenmakers et al., 2016). The modified classification according to Lee and Wagenmakers (2013) was used categorizing 10 < BF_10_ ≥ 30 as “strong evidence for H1,” 3 < BF_10_ ≥ 10 as “moderate evidence for H1,” 1 < BF_10_ ≥ 3, respectively, 0.10 < BF_10_ ≥ 0.33 as “anecdotal evidence for H0“, and BF_10_ = 1 as “no evidence.” For models with at least moderate evidence, indicated by BF_10_, multiple linear regression models (using stepwise backward entry method) were calculated (level of significance α < 0.05). Assumptions of multicollinearity (with Variance Inflation Factor and Tolerance), homoscedasticity, linearity, and normality of residuals (with Q-Q-Plots) and independence of residuals (with Durbin-Watson) were checked for each regression model (Goss-Sampson, 2018). The coefficient of determination *R*^2^_adj_ (adjusted for sample size n and multiple predictors using McNemar’s formula (Goss-Sampson, 2018) was used as indicator for the goodness of model fit for the overall hierarchical multiple linear regression models, and standardized regression weights β as well as *post hoc* Spearman’s rho (ρ) correlation coefficients were determined and tested for significance (level of significance α < 0.05).

Differences between the four walking conditions were calculated for MoCA, ΔTMT, DIA-S, FES-I, MDS-UPDRS III, age, and gender at T1 as well as for all Δwalking parameters. For continuous variables, the Kruskal-Wallis H-test and Dunn’s *post hoc* test (with Bonferroni-Holm-correction for paired comparisons, significant at p_holm_ < 0.05) were used (Goss-Sampson, 2018). For categorical variables, the χ^2^ test was used (Goss-Sampson, 2018). As an additional exploratory analysis, changes of each walking parameter between T1 and T2 were examined for each of the four walking conditions using Wilcoxon signed-ranks test for dependent samples (Goss-Sampson, 2018). Outliers, defined as ±3SD, were excluded for the following parameters: ΔTMT score (n = 2), for ST normal pace step time, stride time, stance time, swing time, DLS, ASYM, ΔASYM, ΔSTV (each n = 1), Δstep time, Δstride time, Δswing time, Δstance time, ΔDLS and ΔDLSV (each n = 2), for ST fast pace gait speed (n = 2) and Δgait speed (n = 2), for DT walking cognitive number of steps, DLSV, STV, ΔDLS and ΔSTV (each n = 1), and Δnumber of steps, Δstride time, Δstance time, and ΔDLSV (each n = 2), and for DT walking-motor number of steps (n = 1).

Data were preprocessed using MATLAB (version 2020b) and Python (version 3.9.1.), and statistical analysis were conducted using JASP (version 0.16.1).

## 3. Results

### 3.1. Descriptive characteristics

A total of *n* = 47 patients with data from the CGA at T1 and the IMU-based movement analysis before and after therapy were included for this analysis (out of those n = 8 did not perform the TMT due to lack of capacity or motivation). Patients were on average 73 years old (SD = 8), 38% were female. Mean number of days between T1 and T2 was 11 days (SD = 3), and patients had on average 10 sessions of physical therapy (SD = 2) and 7 sessions of occupational therapy (SD = 2) during this period. Mean disease duration was 10 years (SD = 8), mean MDS-UPDRS III was 30 points (SD = 14), and median Hoehn and Yahr stage was 3 (IQR = 1) with 60% at stage 3 and 30% at stage 4. Cognitive impairment was previously reported in the medical records in 17% of the cohort, of which 9% were prediagnosed with dementia. The mean MoCA total score was 23 points (SD = 3.7), thus below the cut-off for MCI in patients with PD (see section 2.4.2), and mean ΔTMT score was 86.3 s (SD = 112). Mean total score of the DIA-S was 3 points (SD = 2.2) with 28% of the cohort showing depressive symptoms (cut-off ≥4 points), and mean FES-I total score was 30 points (SD = 11) with 72% of the cohort showing high concern to fall. Patients were comparable over all four walking conditions regarding MoCA total score (H = 0.61, *p* = 0.89), ΔTMT performance (H = 1.75, *p* = 0.63), DIA-S total score (H = 0.94, *p* = 0.82), FES-I total score (H = 1.57, *p* = 0.67), age (H = 0.03, *p* > 0.99), gender (χ^2^ = 0.66, *p* = 0.88), and MDS-UPDRS III total score (H = 6.49, *p* = 0.09). Table 1 provides detailed information of descriptive and clinical characteristics of baseline T1 for all four walking conditions.

**Table 1:** Descriptive characteristics of demographic, clinical and CGA parameters at baseline assessment, days between measurements, therapy sessions, and deltas of walking parameters over all four walking conditions.

Parameters	ST normal pace		ST fast pace		DT walking-cognitive		DT walking-motor	
	*n*	*M* (SD) {Median; IQR}	*n*	*M* (SD) {Median; IQR}	*n*	*M* (SD) {Median; IQR}	*n*	M (SD) {Median; IQR}
Age [years]	47	73 (7.80) {74; 11.5}	32	72 (7.31) {75; 10}	19	72 (9.11) {77; 12}	18	72 (8.96) {77; 12}
Female [n (%)]		18 (38)		11 (34)		7 (37)		5 (28)
Education [years]		10 (1.82)		10 (1.75)		11 (1.80)		10 (1.97)
Days between measurements		11 (2.61)		11 (2.85)		11 (2.95)		11 (3.16)
Physical therapy sessions		10 (2.38)		9 (2.58)		9 (2.96)		8 (3.03)
Occupational therapy sessions		7 (2.11)		7 (2.49)		7 (2.47)		7 (2.69)
Disease duration [years]		10 (7.54)		10 (7.88)		9 (7.97)		8 (6.84)
Hoehn & and Yahr		{3; 1}		{3; 0}		{3; 0}		{3; 0}
LEDD [mg]		721 (349.4)		682 (325.4)		695 (377)		657 (334.5)
MoCA		23 (3.65) {23; 5}		23 (3.4) {23; 5}		24 (3.33) {24; 4.5}		23 (3.93) {23; 5.75)
ΔTMT [s] (8 missing, 2 excluded)		112 (72.9) {86; 79}		123 (75.2) {106; 68.75}		103 (62.4) {83; 44}		98 (64.4) {82.5; 52}
DIA-S^a^		3 (2.16) {2;3}		2 (2.17) {2; 2}		2 (2.40) {2; 2}		2 (2.64) {1.5; 3.5}
FES-I^b^		30 (10.8) {28; 14.5}		28 (8.63) {27.5; 8.5}		27 (7.95) {28; 9}		26 (7.27) {25; 8.5}
MDS-UPDRS III		30 (13.5) {32; 22.5}		28 (13.3) {26.5; 20.25}		23 (13.2) {20; 14}		22 (12.1) {20; 17.25}
Occurrence of dyskinesia [n (%)]		[6; 14]		[4;14]		[1; 6]		[1; 7]
Impact of dyskinesia [n; %]		[3; 6]		[2; 6]		[0; 0]		[0; 0]
Occurrence of FOG^c^		[15; 32]		[9; 28]		[5; 26]		[4; 22]
Walking aid [n (%)]		[11; 23]		[4; 13]		[2; 11]		[0; 0]
Δnumber of steps	47	−0.96 (7.33) {0; 5.50}	32	1.38 (4.92) {1; 4.25}	17	−0.59 (7.92) {1; 5}		−1.89 (7.99) {−2; 13}
Δgait speed [m/s]	46	−0.006 (0.13) {−0.01; 0.13}	31	−0.05 (0.15) {−0.02; 0.20}	19	0.03 (0.19) {0.04; 0.18}		0.04 (0.23) {0.007; 0.28}
Δstep time [s]	45	0.01 (0.07) {0.008; 0.07}	32	0.007 (0.02) {0.008; 0.04}	19	−0.08 (0.16) {−0.06; 0.15}	18	0.0005 (0.07) {0.0004; 0.06}
Δstride time [s]	45	0.05 (0.16) {0.04; 0.16}	32	0.01 (0.05) {0.02; 0.08}	17	−0.10 (0.24) {−0.12; 0.33}		−0.005 (0.14) {0.001; 0.12}
Δswing time [s]	45	−0.003 (0.02) {−0.004; 0.02}	32	−0.004 (0.02) {0.0004; 0.01}	19	−0.02 (0.03) {−0.01; 0.03}		−0.007 (0.02) {−0.007; 0.006}
Δstance time [s]	45	0.05 (0.15) {0.04; 0.16}	32	0.01 (0.05) {0.02; 0.07}	17	−0.09 (0.24) {−0.10; 0.33}		0.003.64 (0.14) {0.01; 0.12}
ΔDLS [s]	45	0.01 (0.06) {0.01; 0.06}	32	0.01 (0.03) {0.01; 0.03}	18	−0.04 (0.12) {−0.04; 0.13}		0.008 (0.079) {0.008; 0.05}
ΔDLSV [s]	45	−0.02 (0.06) {−0.01; 0.03}	32	0.01 (0.04) {0.0003; 0.03	17	−0.05 (0.08) {−0.04; 0.11}		−0.006 (0.10) {−0.007; 0.10}
ΔASYM [s]	46	0.008 (0.04) {0.003; 0.05}	32	−0.0003 (0.03) {−0.0009; 0.03}	19	−0.01 (0.03) {−0.003; 0.04}		−0.0006 (0.04) {−0.007; 0.04}
ΔSTV [s]	46	0.01 (0.04) {−0.02; 0.04}	32	−0.02 (0.08) {0.003; 0.03}	18	−0.06 (0.10) {−0.05; 0.11}		−0.007 (0.10) {−0.005; 0.09}

a Imputed values using individual median imputation for cases with one missing single item; ASYM, asymmetry.

b Imputed values using individualized mean imputation for cases with one or two missing single items.

c Occurrence during measurement; DIA-S, Depression im Alter Scale; DLS, double limb support; DLSV, double limb support variability; DT, dual task; FES-I, Falls Efficacy Scale – International version; FOG, Freezing of gait; IQR, interquartile range; LEDD, levodopa equivalence daily dose (in milligram, mg); M, mean; m/s, meter per seconds; max, maximum; MDS-UPDRS III, Movement Disorder Society-revised version of the motor part of the Unified Parkinson’s Disease Rating Scale; min, minimum; MoCA, Montreal Cognitive Assessment total score; n, sample size; s, seconds; SD, standard deviation; ST, single task; STV, step time variability; Δ, delta of walking parameters as difference of measurements after and before therapy; ΔTMT, delta of Trail Making Test (part B minus part A); %, percentage.

**Figure 1:**
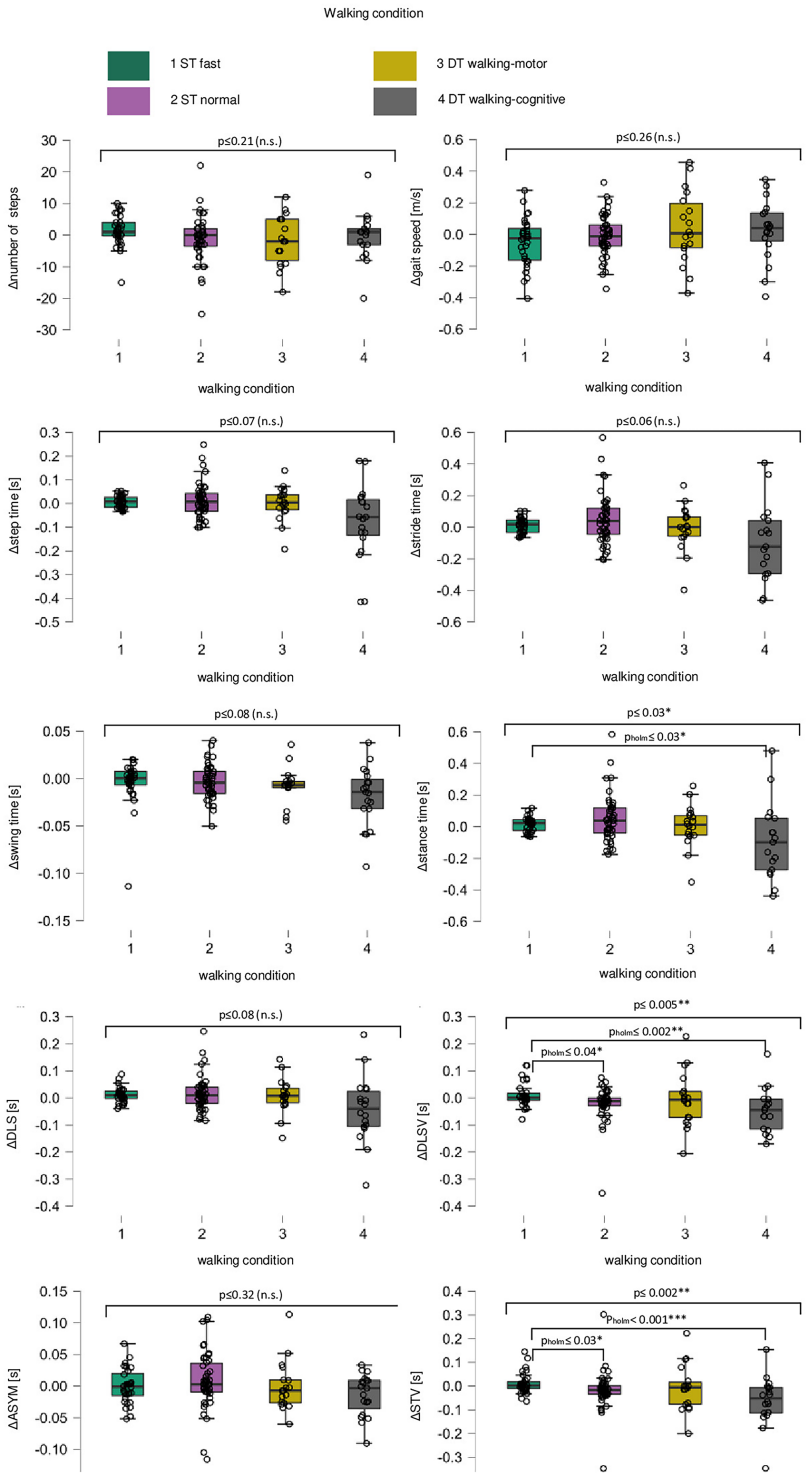
Box plots for change in all walking parameters over all walking conditions. For the change (delta of measurement point T1 minus measurement point T2, Δ) in number of steps, gait speed (meters per second, m/s), step time (seconds, s), stride time (s), swing time (s), stance time (s), double limb support (DLS, s), double limb support variability (DLSV, s), asymmetry (ASYM, s) and step time variability (STV, s), single subject data points (black circles) are given for walking conditions single task fast pace (green, 1), single task normal pace (violet, 2), the dual task walking-motor (yellow, 3) and the dual task walking-cognitive (grey, 4). Between the four walking conditions, differences in the change in walking performance are shown [long black square brackets, using Kruskal–Wallis H-Test, significant differences are marked with *Bonferroni-corrected level of significance *p* ≤ 0.05; **level of significance *p p* ≤ 0.01; non-significant ones are marked with (n.s.)] as well as post-hoc paired group comparisons between each walking condition (short black square brackets, using Dunn’s *post-hoc* test, significant paired-group differences are marked with *Holm-corrected level of significance p_holm_ ≤ 0.05; **level of significance p_holm_ ≤ 0.01, and ***level of significance p_holm_ ≤ 0.001).

For both times, T1 and T2, n = 47 patients successfully completed the 20 m walking distance under ST normal pace, n = 32 under ST fast pace, n = 19 under DT walking-cognitive, and n = 18 under DT walking-motor. As not all subjects were capable to participate in every condition, the sample size decreased with capacity-dependent task prioritization and increasing demands per condition (e.g., not necessarily all subjects who performed the ST normal pace condition could also perform ST fast pace due to reduced physical capacity). For the three walking conditions that were performable with a walking aid, 11% (DT walking-cognitive) to 23% (ST normal pace) of the patients used their walking aid. Differences between the four walking conditions for all Δwalking parameters as well as paired group comparisons between the conditions are illustrated in Figure 1. Results of the additional exploratory comparisons of all Δwalking parameters for each walking condition between T1 and T2 are shown in Supplementary Table 1, significant differences after treatment are illustrated in Supplementary Figure 1.

### 3.2. Regression analyses

**Figure 2:**
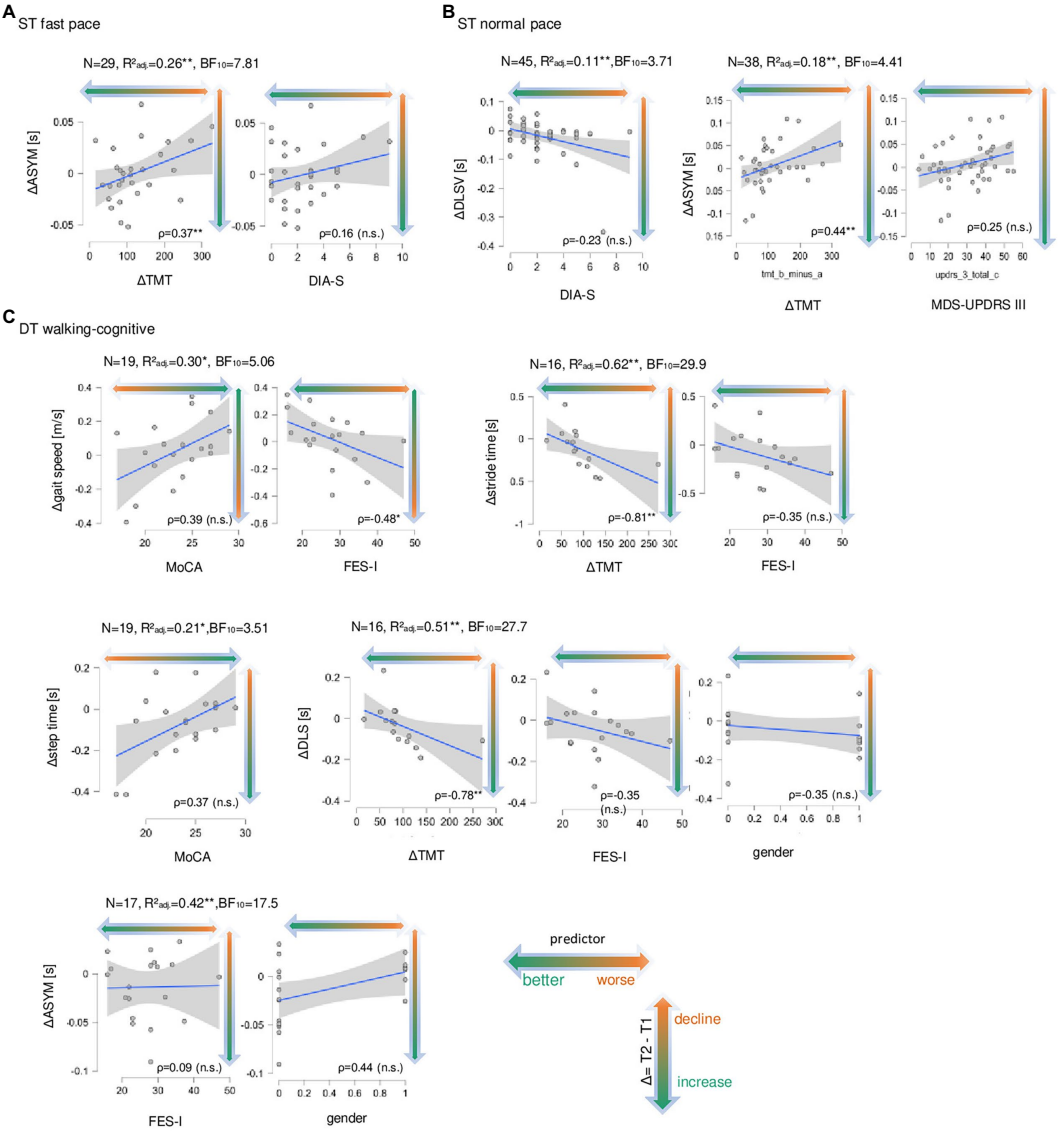
Correlation plots of the significant Regression models with the relevant model predictors for change in walking parameters. In **(A)** for single task fast pace walking condition (ST fast pace) the change (delta of measurement point T1 minus measurement point T2, Δ) in asymmetry (ASYM, in seconds, s) is shown on the ordinates, the delta of Trail Making Test (part B minus part A, ΔTMT, s) and the total score of the Depression im Alter-Scale (DIA-S) are on the abscissas. Sample size N is given as well as the adjusted coefficient of determination *R*^2^_adj_, Bayes factor BF_10_ and Spearman’s rank correlation (ρ) between ΔASYM and ΔTMT and between ΔASYM and DIA-S, significant correlation coefficients are marked with * (level of significance *p* ≤ 0.05) and ** (level of significance *p p* ≤ 0.01), non-significant ones are marked with (n.s.). Data points (gray dots), regression lines with confidence intervals (blue lines with surrounding gray boxes) are shown for the Δwalking parameter. The colored direction of the arrow indicated the direction of the change in walking parameters from T2 to T1 from longer/slower (orange) to shorter/faster (green). In **(B)** the same is shown for single task normal pace walking condition for the change in double limb support variability (ΔDLSV,s) with DIA-S as well as for ΔASYM (S) and ΔTMT and the total score of the Movement Disorder Society-revised version of the motor part of the Unified Parkinson’s Disease Rating Scale (MDS-UPDRS III). In **(C)** the same is shown for DT motor-cognitive walking condition (DT walking-cognitive) for Δgait speed (in meter per seconds, m/s) and the total score of the Montreal cognitive Assessment (MoCA), for Δstride time (s) and ΔTMT and the total score of the Falls-Efficacy-Scale-International version (FES-I), for Δstep time (s) and MoCA, for Δdouble limb support (DLS, s) and ΔTMT, FES-I and gender (“1,” female) as well as for ΔASYM (s), and FES-I and gender.

The main results of Bayesian and multiple linear regression analyses for the cognitive and affective parameters as independent variables and Δwalking parameters as dependent variables are summarized here and illustrated in Figure 2, while Table 2 provides more detailed information for all significant regression models. Under ST fast pace condition, Bayesian regression suggested moderate evidence in favor of the model with ΔASYM (BF_10_ = 7.81), and ΔTMT and DIA-S. The overall backward multiple linear regression model was significant (*p* = 0.008) with a coefficient of determination of *R*^2^_adj_ = 26%. The effect was driven by ΔTMT (β = 0.56, *p* = 0.004) with a moderately positive *post hoc* correlation (*ρ* = 0.37, *p* = 0.009, Figure 2A) and DIA-S (β = 0.44, *p* = 0.02) with no significant *post hoc* correlation (*ρ* = 0.16, *p* = 0.37, Figure 2A). This means, there is moderate evidence that, together, ΔTMT and DIA-S significantly explain about one-quarter of the variance of ΔASYM, and that higher positive values of ΔASYM seem to be associated with higher ΔTMT and DIA-S scores. Furthermore, Bayesian regression suggested anecdotal evidence for H0 with regard to all other Δwalking parameters indicating no association with any of the independent variables in this cohort (all BF_10_ < 3; all p’s > 0.05). Therefore, individual effects for these parameters were not further interpreted and are not shown here.

**Table 2:** Backward multiple linear regression models and Bayes factors for significant deltas of walking parameters.

Dependent variables	Independent variables found as predictors	*n*	*R* ^2^_adj._	*F*	BF_10_	*β*	*p*
*ST fast pace*							
ΔASYM [s]^2^		29	0.26	5.9	7.81^a^		0.008**
	ΔTMT					0.56	0.004**
	DIA-S					0.44	0.02*
*ST normal pace*							
Δstep time [s]^2^		37	0.14	6.85	4.49^a^		0.01**
	MDS-UPDRS III					0.4	0.01**
Δstride time [s]^2^		37	0.16	7.7	6.50^a^		0.009**
	MDS-UPDRS III					0.42	0.009**
Δstance time [s]^1^		45	0.11	6.26	3.18^a^		0.02*
	MDS-UPDRS III					0.36	0.02*
ΔDLS [s]^1^		45	0.11	6.51	3.55^a^		0.01**
	MDS-UPDRS III					0.36	0.01**
ΔDLSV [s]^1^		45	0.11	6.6	3.71^a^		0.01**
	DIA-S					−0.36	0.01**
ΔASYM [s]^2^		38	0.18	5.41	4.41^a^		0.009**
	ΔTMT					0.34	0.03*
	MDS-UPDRS III					0.28	0.08
*DT walking-cognitive*							
Δgait speed [s]^1^		19	0.3	4.94	5.06^a^		0.02*
	MoCA					0.42	0.05*
	FES-I					−0.41	0.06
Δstep time [s]^1^		19	0.21	5.66	3.51^a^		0.03*
	MoCA					0.5	0.03*
Δstride time [s]^2^		16	0.62	9.16	29.9^b^		0.002**
	ΔTMT					−0.48	0.01**
	FES-I					−0.4	0.03*
	Gender					−0.38	0.05*
ΔDLS [s]^2^		16	0.51	6.19	27.7^b^		0.009**
	ΔTMT					−0.46	0.03*
	FES-I					−0.4	0.06
	Gender					−0.32	0.12
ΔASYM [s]^2^		17	0.42	6.68	17.5^b^		0.009**
	FES-I					−0.47	0.03*
	Gender					0.69	0.004**

a Moderate evidence for H1; ASYM, asymmetry.

b Strong evidence for H1; BF10, Bayes factor as measure for strength of model evidence; DIA-S, Depression im Alter Scale; DLS, double limb support; DLSV, double limb support variability; DT, dual task; *F*, test statistic from ANOVA used for testing significance of the multiple regression models; m/s, meter per seconds; MDS-UPDRS III, Movement Disorder Society-revised version of the motor part of the Unified Parkinson’s Disease Rating Scale; MoCA, Montreal Cognitive Assessment total score; *n*, sample size; *p* ≤ 0.05*, significant on level of significance α ≤ 0.05; *p* ≤ 0.01**, significant on level of significance α ≤ 0.01; *R*^2^_adj_., multiple regression coefficient adjusted for sample size and number of model parameters; s, seconds; ST, single task; *β*, standardized regression weights; Δ, delta of walking parameters as difference of measurements after and before therapy; ΔTMT, delta of Trail Making Test (part B minus part A).

1 Model included MoCA, DIA-S, FES-I, age, gender, walking aid, and MDS-UPDRS III.

2 Model included ΔTMT, DIA-S, FES-I, age, gender, walking aid, and MDS-UPDRS III.

Under ST normal pace, Bayesian regression suggested moderate evidence for ΔASYM (BF_10_ = 4.41) with ΔTMT and MDS-UPDRS III included (Table 2). The overall backward multiple linear regression model was significant (*p* = 0.009, *R*^2^_adj_ = 18%), mainly driven by the ΔTMT (β = 0.34, *p* = 0.03) with a moderately positive *post hoc* correlation (*ρ* = 0.44, *p* = 0.008, Figure 2B), and to less extent by the MDS-UPDRS III (β = 0.28, *p* = 0.08) with no significant *post hoc* correlation (*ρ* = 0.25, *p* = 0.11, Figure 2B). This means, that, together, ΔTMT and MDS-UPDRS III explain nearly one-fifth of the variance of ΔASYM, and that higher values of ΔASYM are associated with higher ΔTMT and MDS-UPDRS III scores. When the MoCA total score was included in the model, there was moderate evidence for ΔDLSV (BF_10_ = 4.41) with DIA-S included (Table 2). The overall backward multiple linear regression model was significant with *R*^2^_adj_ = 11%, driven by a negative effect of the DIA-S (β = −0.36, *p* = 0.01), but there was no significant *post hoc* correlation (*ρ* = −0.23, *p* = 0.12, Figure 2B).

Under DT walking-cognitive, Bayesian regression suggested moderate evidence for Δgait speed (BF_10_ = 5.06) with MoCA and FES-I included (Table 2). The overall backward multiple linear regression model was significant (*p* = 0.02) with *R*^2^_adj_ = 30%. The effect was mainly driven by a positive effect of MoCA (β = 0.42, *p* = 0.05) with a non-significant moderately positive *post hoc* correlation (*ρ* = 0.39, *p* = 0.10, Figure 2C) and to less extent by a negative effect of FES-I (β = −0.41, *p* = 0.06) with a significant moderately negative *post hoc* correlation (*ρ* = −0.48, *p* = 0.04, Figure 2C). This implies, that, together, total scores of MoCA and FES-I explain nearly one-third of the variance of Δgait speed, and that lower values of Δgait speed are associated with lower MoCA scores and higher FES-I scores. Also, there was moderate evidence for Δstep time (BF_10_ = 3.51) with the MoCA included (Table 2). The overall backward multiple linear regression model was significant (*p* = 0.03) with *R*^2^_adj_ = 21%, driven by a positive effect of the MoCA (β = 0.50, *p* = 0.002) with a non-significant moderately positive *post hoc* correlation (*ρ* = 0.37, *p* = 0.11, Figure 2C), implying moderate evidence for the MoCA total score explains about one-fifth of the variance of Δstep time, and lower values of Δstep time seem to be associated with lower MoCa total scores. In case the ΔTMT was included in the model, Bayesian regression suggested strong evidence for Δstride time (BF_10_ = 29.90) and with ΔTMT, FES-I and gender included (Table 2). The overall backward multiple linear regression model was significant for Δstride time (*p* = 0.002) with *R*^2^_adj_ = 62%, driven by negative effects of ΔTMT (β = −0.48, *p* = 0.01), FES-I (β = −0.40, *p* = 0.03) and gender (β = −0.38, *p* = 0.05 (i.e., women have lower values in Δstride time than men). There were significant strongly negative *post hoc* correlations between Δstride time and ΔTMT (*ρ* = −0.81, *p* = 0.0004, Figure 2C) and gender (*ρ* = −0.51, *p* = 0.04, Figure 2C) and a non-significant moderately negative *post hoc* correlation with FES-I (*ρ* = −0.35, *p* = 0.17, Figure 2C). Also, there was strong evidence for ΔDLS (BF_10_ = 27.73) with ΔTMT, FES-I and gender included (Table 2). The overall backward multiple linear regression model was significant for ΔDLS (*p* = 0.009) with *R*^2^_adj_ = 51%, driven by negative effects of ΔTMT (β = −0.46, *p* = 0.03) with a significant strongly negative *post hoc* correlation (*ρ* = −0.78, *p* = 0.0004, Figure 2C), and to less extent by negative effects of FES-I (β = −0.40, *p* = 0.06), and gender (β = −0.32, *p* = 0.12) with non-significant moderately negative *post hoc* correlations for FES-I (*ρ* = −0.35, *p* = 0.17, Figure 2C) and gender (*ρ* = −0.38, *p* = 0.12, Figure 2C). This implies with strong evidence that the ΔTMT, together with FES-I total score and gender, explains nearly two-thirds of the variance of Δstride time as well as half of the variance of ΔDLS. Thus, lower values of Δstride time and ΔDLS are associated with higher values of ΔTMT and FES-I scores and female gender. Furthermore, Bayesian regression suggested strong evidence for ΔASYM (BF_10_ = 17.51) with FES-I and gender included (Table 2). The overall backward multiple linear regression model was significant for ΔASYM (*p* = 0.009) with *R*^2^_adj_ = 42%. The effect was mainly driven by a positive gender effect (β = 0.69, *p* = 0.004, i.e., woman have higher values in ΔASYM than men) with a nearly significant moderately positive *post hoc* correlation (*ρ* = 0.44, *p* = 0.06, Figure 2C), and by a negative effect of FES-I (β = −0.47, *p* = 0.03) with no significant *post hoc* correlation (*ρ* = 0.09, *p* = 0.71, Figure 2C). Under DT walking-motor Bayesian regression suggested only anecdotal evidence for H0 for all Δwalking parameters indicating no association with the parameters of the extended CGA in this cohort (BF_10_ < 3; all *p*’s > 0.05). Therefore, individual effects were not further interpreted.

## 4. Discussion

In our study, we assessed cognitive and affective non-motor symptoms (namely global cognitive performance, EF and divided attention, depressive symptoms and FOF) as well as walking performance rehabilitation in acutely hospitalized in patients with advanced PD. The aim of this study was to investigate the relationship between these non-motor symptoms at admission and the change in walking performance under ST and DT conditions after 2 weeks of individualized early rehabilitation. The main result of our study is that cognitive performance at admission can predict the change of walking performance during the hospital stay. Particularly, reduced EF and divided attention should be considered as limiting factors for treatment success, especially in situations with additional cognitive demand while walking.

In summary, under both ST walking conditions, the results of the regression analyses show a ceiling effect with regard to patients with higher performance in EF and divided attention that showed higher reduction of ASYM after treatment. This is also consistent with a comparative study showing that both individuals with PD and idiopathic fallers show a more asymmetric walking pattern under normal ST walking conditions than older healthy individuals and that under additional attention demand (in the sense of DT) ASYM is even more pronounced in these former two groups (Yogev et al., 2007). As ASYM is also known to be associated with higher PD disease severity (Polhemus et al., 2021; Vila et al., 2021), these findings suggest that patients with higher capacity in EF and divided attention seem to be able to better compensate for their asymmetric gait pattern after treatment.

Under DT walking cognitive condition, patients with lower performance in EF and divided attention showed more reduced stride time and DLS after treatment than patients with better performance in EF and divided attention. This reduction might reflect the acute medical indication with which the patients were admitted to the clinic and the advanced stage of the disease, which is associated with more rapid progression and more severe symptom fluctuations. However, while stride time and gait speed are readily interpretable for clinical aspects (e.g., lower gait speed and stride time is known to be associated with higher PD disease severity (Polhemus et al., 2021), the stand-alone practical meaning and clinical utility as well as the expectable responsiveness to treatment of other parameters, such as DLSV, remains inconsistent throughout the literature (Bouça-Machado et al., 2020; Polhemus et al., 2021). Accordingly, considering the whole walking profile of our cohort (reduction of step time, stride time, and DLS while walking slower in this DT walking-cognitive condition), patients with lower performance in EF and divided attention appear to walk more cautiously after treatment and compared to patients with better performance in EF and divided attention, especially under DT with additional cognitive demand. On the other hand, patients with higher global cognitive performance had an increased gait speed, but showed also less reduction in step time, while patients with lower global cognitive performance showed more decreased gait speed after treatment. This association between lower global cognitive performance and reduced gait speed in PD was also found under ST in another recent cross-sectional study in a cohort of people with PD (comparable to our cohort with regard to age, global cognitive performance also measured with the MoCA and severity of motor symptoms measured with the MDS-UPDRS III but not acutely hospitalized) (Shearin et al., 2021). Furthermore, similar results were obtained in a study on targeted DT training for patients with PD, where patients with lower cognitive performance also showed a lower increase in gait speed (Strouwen et al., 2019). In addition, a higher level of depressive symptoms and more severe motor symptoms contributed to less extent, but in the same direction, to the change in DLSV and ASYM under ST walking conditions, and the same was true for higher FOF and gender with regard to the change in gait speed, stride time and DLS, under DT walking-cognitive condition. The more complex DT walking conditions could not be performed by more severely affected patients in our cohort. Therefore, further investigations are needed to validate the results of this study.

Overall, the results of the regression analyses show a ceiling effect in patients with higher performance in EF and divided attention as well as higher global cognitive performance, and with lower FOF, less depressive symptoms and less severe motor symptoms change less in their walking performance after ERGCT. This is comparable with results of another study in patients with advanced PD that identified better mental state as determinant factor for long-term treatment effects of physical therapy training on physical activity (Nieuwboer et al., 2002).

The additional analysis performed in our study regarding significant differences in walking performance between the acute medical admission and at the end of the 2-week treatment also indicated an overall tendency to decrease swing time in both DT conditions. In addition, although there were no significant changes in spatio-temporal walking parameters in both DT conditions as well as in walking with normal pace, there was also no deterioration in walking performance. Furthermore, when forced to walk in a fast pace, patients tend to show an increased number of steps and DLS after ERGCT, while their gait speed remains similar after treatment. Because fast walking corresponds to a higher level of motor difficulty, the results suggest that this group of patients cannot adequately compensate for the problems associated with faster walking. A comparison of treatment effects between the different walking conditions showed a significantly greater reduction in stance time, DLSV, and STV under DT than under ST. This finding might be explained by the described positive effect of external cueing in patients with PD, where the respective additional task corresponds to a rhythm generator (Ginis et al., 2017). Future studies with larger cohorts should consider this aspect in a detailed and sufficient individualized treatment protocol.

Therefore, our results indicate that 2 weeks of ERGT can modulate walking impairment of advanced PD in acutely hospitalized patients. However, pre-existing cognitive impairments, especially deficits in EF and divided attention seem to limit this effect. Therefore, for patients with pronounced cognitive and affective non-motor symptoms (i.e., high level of depressive symptoms and FOF), a different therapeutic framework than ERGT may be required to adequately address these symptoms and their influence on walking impairments. To our best knowledge, this is the first study to analyze the change of several IMU-based spatio-temporal walking parameters after this sort of treatment in this vulnerable cohort with regard to the impact of pre-existing non-motor symptoms on treatment success. Other studies focused on standardized training protocols for such as DT training or external cueing (Ginis et al., 2017; Geroin et al., 2018; Strouwen et al., 2019), used longer intervention intervals and settings other than early rehabilitation (Nieuwboer et al., 2002; Ginis et al., 2017; Strouwen et al., 2017; Geroin et al., 2018; Serrao et al., 2019; Strouwen et al., 2019; Bouça-Machado et al., 2021), excluded patients with advanced PD and/or cognitive impairment (Nieuwboer et al., 2002; Ginis et al., 2017; Strouwen et al., 2017; Geroin et al., 2018; Serrao et al., 2019; Strouwen et al., 2019; Bouça-Machado et al., 2021), did not examine DT walking conditions (Serrao et al., 2019), and mainly calculated group comparisons between different training groups or individuals with PD and healthy controls, which addresses different scientific questions (Vervoort et al., 2016; Ginis et al., 2017; Geroin et al., 2018; Serrao et al., 2019; Bouça-Machado et al., 2021). There is currently insufficient knowledge about this and, accordingly, there are no specific recommendations in rehabilitation guidelines nor sufficient information of treatment efficacy (Dietrichs and Odin, 2017; Nonnekes and Nieuwboer, 2018).

## 5. Limitations

First, acute factors of illness (e.g., infections, worsening of PD or other symptoms, and recent fall events.) may influence the overall condition of the patients, which was not controlled for in this study. However, due to the requirement of special attention in treatment along with their health condition, we argue that a specific investigation of this vulnerable cohort is justified. Second, due to capacity reasons and increased motor difficulty of the tasks, the number of participants decreased with increasing difficulty of the tasks. Therefore, more severely affected patients may not have performed more complex walking tasks and so the comparability of the tasks is limited. Furthermore, due to the integration into a comprehensive movement protocol (Geritz et al., 2020) randomization of the tasks was not possible for reasons of feasibility and error reduction during the examination. We argue that the decreased subject number for successful performance in tasks with higher cognitive and motor complexity can be taken as an additional indication that patients with advanced PD can less likely master those complex (but still required in everyday life) demands. Third, reporting detailed information about the content of single sessions of therapy was not possible here, as the study was implemented in a clinical routine of a neurogeriatric ward for acute and early rehabilitation according to personalized treatment plans based on the patients’ needs. Therefore, the adaption of the content of the therapeutic sessions was not intended in the study protocol (Geritz et al., 2020) and no specific statements regarding treatment efficacy are possible from this analysis. Nonetheless, skilled individualized treatment is in our point of view a most sufficient way to address the needs of this vulnerable cohort in everyday medical care and should therefore be a point of focus in treatment studies. Fourth, patients with walking aids, FOG, and dyskinesia were also included, but a more granular analysis of their influence on change in walking performance was not possible due to the small sample size. We still included these patients since they are a common part of our neurogeriatric PD cohort and to increase the sample size as much as possible. Future studies should focus on these aspects specifically with larger cohorts of patients with advanced PD. Fifth, in order to collect motor data while patients were at their best possible motor condition, patients were tested during the medication “ON” state. Therefore, no conclusions regarding the non-medicated (“OFF”) status can be drawn from these analyses. Sixth, this study assesses general cognitive performance, as well as EF and divided attention; however, it should be noted that other specific cognitive domains were not assessed. Finally, neither healthy control subjects nor age-matched inpatients with other diseases as controls were included at this stage, which would provide more direct conclusions regarding pathology-specific aspects as well as differences in treatment efficacy.

## 6. Conclusion

This study provides new insights regarding the influential value of cognitive and affective non-motor symptoms for the change in spatio-temporal walking parameters in acutely hospitalized patients with advanced PD after 2 weeks early geriatric rehabilitation. Therefore, these results help close a gap in knowledge regarding relevant characteristics of this vulnerable group of patients, that need to be considered for planning and prognosis of individualized treatment of walking performance. There is evidence that especially EF and divided attention (and global cognitive performance, together with FOF, depressive symptoms and severe motor symptoms) can be associated with change in walking performance (in particular ASYM and gait speed) under both ST and DT walking conditions. After treatment, patients with advanced PD and higher performance in EF and divided attention show reduced ASYM under ST. On the other hand, patients with lower performance in EF and divided attention in this cohort seem to have a more cautious walking pattern (characterized by reduced step time, stride time, and DLS while walking slower) when an additional cognitive task requires to split attention, while there is a ceiling effect for patients, that are less affected by deficits in global cognition, EF and divided attention, depressive symptoms and FOF. This might be a protective aspect with regard to the acute medical condition and the expected progression of walking problems without treatment at this stage of the disease. Thus, for the implementation of individualized multimodal care in an early rehabilitative neurogeratric setting, it remains essential to consider cognitive and affective non-motor symptoms. Future studies need to take these factors into account and should focus on the development of algorithms to address the individual needs required due to differences in non-motor characteristics in an evidence-based manner. Furthermore, our results indicate that even a short-term early geriatric rehabilitation can help to delay the progression of walking disabilities in acutely hospitalized patients with advanced PD. Therefore, it is an essential brick in the treatment concept for this vulnerable patient group and their complex disease.

## Data availability statement

The raw data supporting the conclusions of this article will be made available by the authors, without undue reservation.

## Ethics statement

The studies involving human participants were reviewed and approved by Ethics committee of the Medical Faculty of the Christian-Albrechts-University of Kiel. The patients/participants and, if applicable, their legal representatives, provided their written informed consent to participate in this study.

## Author contributions

JG and WM made substantial contributions to the conception and design of all parts of the study, trained and supervised the examiners, and were responsible for acquisition, analysis, and interpretation of data and in drafting as well as revising the manuscript. JW, CH, MH, and ME made substantial contributions to the conception and design, training, and supervision of the examiners. NB made substantial contributions to the conception and design, analysis and interpretation of the data. CM, together with JG, JW, and CH, was responsible for the implementation organization of data. MH, ME, JK, and HK made substantial contributions to the conception and design, and to acquisition regarding clinical data for their involved Department of Neurology in Kiel (Germany). All authors revised the manuscript critically for important intellectual content. All authors have given their final approval of the version to be published. Each author has participated sufficiently in the work and takes public responsibility for appropriate portions of the content and agrees to be accountable for all aspects of the work in ensuring that questions related to the accuracy or integrity of any part of the work are appropriately investigated and resolved.

## Funding

The study does not receive any external funding. We acknowledge financial support by DFG within the funding programme Open Access-Publikationskosten.

## Conflict of interest

The authors declare that the research was conducted in the absence of any commercial or financial relationships that could be construed as a potential conflict of interest.

## Publisher’s note

All claims expressed in this article are solely those of the authors and do not necessarily represent those of their affiliated organizations, or those of the publisher, the editors and the reviewers. Any product that may be evaluated in this article, or claim that may be made by its manufacturer, is not guaranteed or endorsed by the publisher.

## Abbreviations

ASYM, Mean step time asymmetry
ComOn, Cognitive and Motor Interaction in the Older Population
DIA-S, *Depression im Alter* Scale
DLS, Mean double limb support
DLSV, Mean double limb support variability
DT, Dual task
ERGCT, Early rehabilitative geriatric complex therapy
FES-I, Falls Efficacy Scale – International version
FOF, Fear of Falling
FOG, Freezing of gait
LEDD, Levodopa equivalence daily dose
MCI, Mild Cognitive Impairment
MoCA, Montreal Cognitive Assessment
PD, Parkinson’s Disease
ST, Single task
STV, Mean step time variability
TMT, Trail Making Test
MDS UPDRS, Revised version of the Unified Parkinson’s Disease Rating Scale
