# Supplementary figures and images for: Cognitive parameters can predict change of walking performance in advanced Parkinson’s disease – Chances and limits of early rehabilitation
Source: Front Aging Neurosci. 2022 Dec 22;14:1070093. doi: 10.3389/fnagi.2022.1070093 (PMC9813446; doi:10.3389/fnagi.2022.1070093)

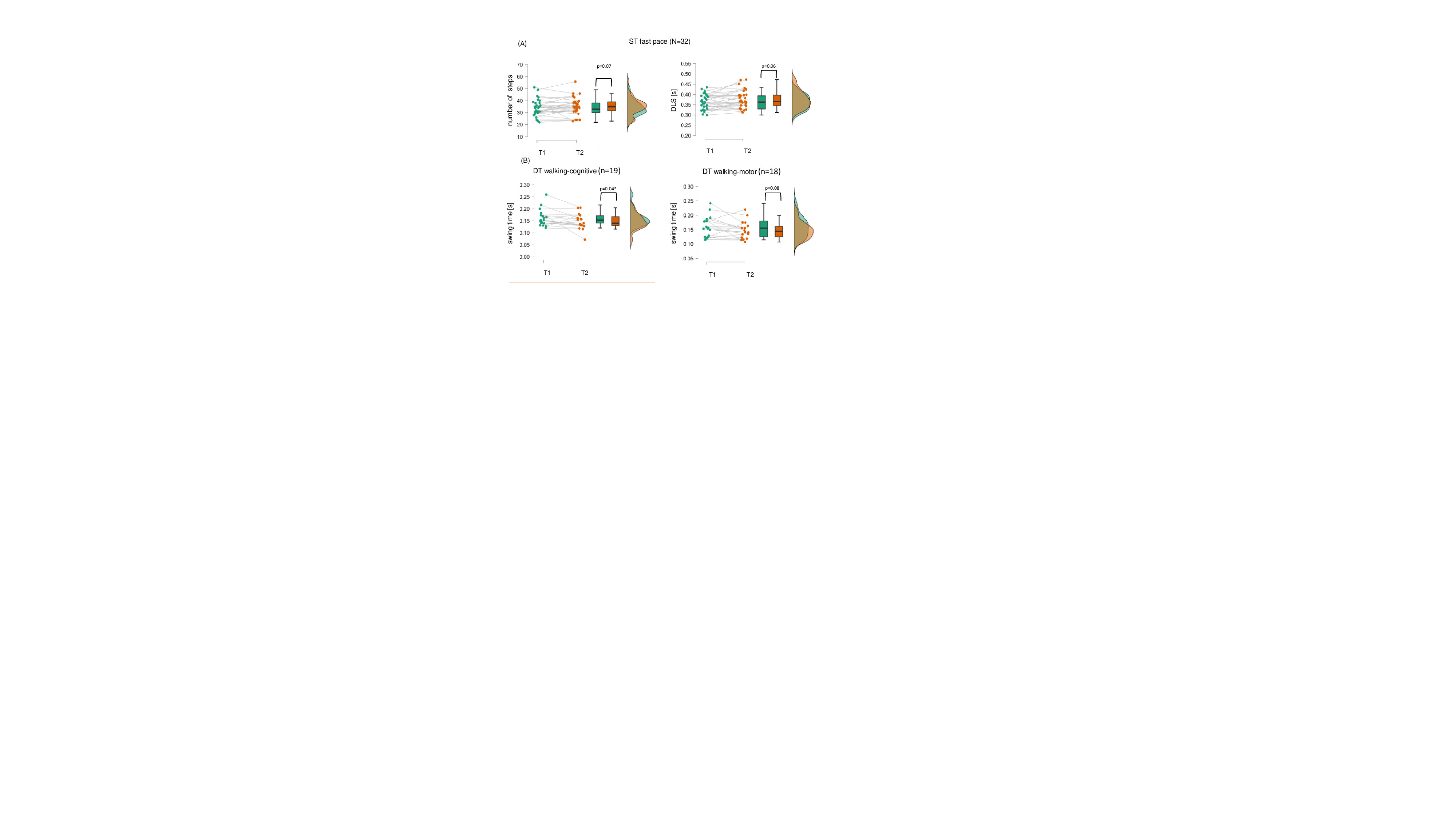

Supplement: Supplementary file 1 [file Image_1.JPEG]
