# Supplementary material for: Cognitive parameters can predict change of walking performance in advanced Parkinson’s disease – Chances and limits of early rehabilitation
Source: Front Aging Neurosci. 2022 Dec 22;14:1070093. doi: 10.3389/fnagi.2022.1070093 (PMC9813446; doi:10.3389/fnagi.2022.1070093)
Supplement: Supplementary file 2 [file Table_1.DOCX]

Suppl.-Tab. 1: Explorative descriptive comparisons for change in walking parameters between T1 and T2 for all four walking conditions

|  | **ST Normal pace** | | | | | | | | **ST fast Pace** | | | | | | | **DT walking-cognitive** | | | | | | | **DT walking-motor** | | | | | | |
| --- | --- | --- | --- | --- | --- | --- | --- | --- | --- | --- | --- | --- | --- | --- | --- | --- | --- | --- | --- | --- | --- | --- | --- | --- | --- | --- | --- | --- | --- |
|  |  | **n** | **M** | **SD** | **Median** | **IQR** | **W** | **p** | **n** | **M** | **SD** | **Median** | **IQR** | **W** | **p** | **n** | **M** | **SD** | **Median** | **IQR** | **W** | **p** | **n** | **M** | **SD** | **Median** | **IQR** | **W** | **p** |
| number of steps | T1 | 47 | 39.1 | 10.4 | 37 | 8.50 | 502 | 0.36 | 32 | 34 | 6.84 | 33 | 8 | 105 | 0.07 | 18 | 38.9 | 8.47 | 39 | 12 | 76 | 0.70 | 17 | 42.18 | 10.29 | 42 | 16 | 95 | 0.40 |
|  | T2 | 47 | 38.2 | 7.63 | 38 | 7.50 |  |  | 32 | 35.3 | 7.32 | 35 | 7.25 |  |  | 18 | 38.6 | 8.37 | 38 | 12.75 |  |  | 17 | 40.29 | 8.95 | 40 | 10 |  |  |
| gait speed [m/s] | T1 | 47 | 0.78 | 0.20 | 0.78 | 0.30 | 549 | 0.93 | 30 | 0.98 | 0.18 | 0.96 | 0.27 | 280 | 0.34 | 19 | 0.76 | 0.25 | 0.72 | 0.35 | 70 | 0.33 | 18 | 0.73 | 0.28 | 0.65 | 0.36 | 70 | 0.52 |
|  | T2 | 47 | 0.79 | 0.22 | 0.77 | 0.24 |  |  | 31 | 0.97 | 0.24 | 0.94 | 0.29 |  |  | 19 | 0.79 | 0.27 | 0.79 | 0.31 |  |  | 18 | 0.77 | 0.28 | 0.73 | 0.28 |  |  |
| step time [s] | T1 | 46 | 0.59 | 0.11 | 0.56 | 0.09 | 517 | 1.00 | 32 | 0.49 | 0.05 | 0.49 | 0.07 | 176 | 0.16 | 19 | 0.62 | 0.15 | 0.60 | 0.13 | 127 | 0.21 | 18 | 0.61 | 0.14 | 0.59 | 0.17 | 102 | 0.50 |
|  | T2 | 46 | 0.59 | 0.10 | 0.57 | 0.10 |  |  | 32 | 0.50 | 0.06 | 0.49 | 0.08 |  |  | 19 | 0.59 | 0.09 | 0.59 | 0.12 |  |  | 18 | 0.58 | 0.08 | 0.57 | 0.10 |  |  |
| stride time [s] | T1 | 46 | 1.18 | 0.21 | 1.11 | 0.18 | 510 | 0.94 | 32 | 0.98 | 0.10 | 0.98 | 0.14 | 178 | 0.17 | 19 | 1.24 | 0.30 | 1.19 | 0.25 | 127 | 0.21 | 18 | 1.21 | 0.28 | 1.17 | 0.34 | 102 | 0.50 |
|  | T2 | 46 | 1.18 | 0.19 | 1.12 | 0.19 |  |  | 32 | 1.00 | 0.11 | 0.97 | 0.17 |  |  | 19 | 1.18 | 0.18 | 1.17 | 0.25 |  |  | 18 | 1.16 | 0.16 | 1.13 | 0.20 |  |  |
| swing time [s] | T1 | 46 | 0.15 | 0.03 | 0.14 | 0.03 | 542 | 0.79 | 32 | 0.13 | 0.02 | 0.13 | 0.02 | 261 | 0.96 | 19 | 0.16 | 0.03 | 0.15 | 0.03 | 147 | 0.04* | 18 | 0.16 | 0.04 | 0.16 | 0.05 | 126 | 0.08 |
|  | T2 | 46 | 0.15 | 0.02 | 0.14 | 0.03 |  |  | 32 | 0.13 | 0.02 | 0.12 | 0.02 |  |  | 19 | 0.15 | 0.03 | 0.14 | 0.04 |  |  | 18 | 0.15 | 0.03 | 0.14 | 0.04 |  |  |
| stance time [s] | T1 | 46 | 1.03 | 0.18 | 0.97 | 0.14 | 519 | 0.99 | 32 | 0.86 | 0.09 | 0.85 | 0.15 | 179 | 0.11 | 19 | 1.08 | 0.26 | 1.03 | 0.21 | 125 | 0.24 | 18 | 1.05 | 0.25 | 1.00 | 0.28 | 9 | 0.58 |
|  | T2 | 46 | 1.03 | 0.17 | 0.98 | 0.18 |  |  | 32 | 0.88 | 0.10 | 0.85 | 0.14 |  |  | 19 | 1.03 | 0.15 | 1.04 | 0.20 |  |  | 18 | 1.01 | 0.14 | 0.99 | 0.21 |  |  |
| DLS [s] | T1 | 46 | 0.44 | 0.08 | 0.42 | 0.06 | 538 | 0.82 | 32 | 0.36 | 0.04 | 0.36 | 0.06 | 163 | 0.06 | 19 | 0.46 | 0.12 | 0.44 | 0.09 | 105 | 0.71 | 18 | 0.45 | 0.11 | 0.42 | 0.12 | 86 | 1.00 |
|  | T2 | 46 | 0.44 | 0.07 | 0.42 | 0.08 |  |  | 32 | 0.38 | 0.04 | 0.37 | 0.05 |  |  | 19 | 0.44 | 0.06 | 0.45 | 0.08 |  |  | 18 | 0.43 | 0.06 | 0.42 | 0.09 |  |  |
| DLSV [s] | T1 | 47 | 0.05 | 0.05 | 0.04 | 0.03 | 621 | 0.25 | 32 | 0.03 | 0.02 | 0.03 | 0.02 | 221 | 0.43 | 18 | 0.08 | 0.07 | 0.06 | 0.03 | 110 | 0.30 | 18 | 0.07 | 0.07 | 0.05 | 0.05 | 83 | 0.78 |
|  | T2 | 45 | 0.04 | 0.02 | 0.03 | 0.02 |  |  | 32 | 0.05 | 0.04 | 0.03 | 0.04 |  |  | 19 | 0.05 | 0.03 | 0.04 | 0.02 |  |  | 17 | 0.07 | 0.08 | 0.05 | 0.05 |  |  |
| ASYM [s] | T1 | 45 | 0.04 | 0.02 | 0.03 | 0.03 | 485 | 0.72 | 32 | 0.03 | 0.02 | 0.02 | 0.04 | 273 | 0.88 | 19 | 0.04 | 0.02 | 0.03 | 0.04 | 104 | 0.74 | 18 | 0.04 | 0.03 | 0.03 | 0.05 | 56 | 0.21 |
|  | T2 | 47 | 0.04 | 0.04 | 0.03 | 0.04 |  |  | 32 | 0.03 | 0.03 | 0.03 | 0.02 |  |  | 19 | 0.04 | 0.03 | 0.03 | 0.02 |  |  | 18 | 0.05 | 0.04 | 0.04 | 0.06 |  |  |
| STV [s] | T1 | 47 | 0.06 | 0.05 | 0.05 | 0.03 | 613 | 0.29 | 32 | 0.04 | 0.02 | 0.04 | 0.03 | 208 | 0.30 | 18 | 0.09 | 0.07 | 0.07 | 0.06 | 123 | 0.11 | 18 | 0.08 | 0.08 | 0.07 | 0.04 | 84 | 0.75 |
|  | T2 | 45 | 0.05 | 0.02 | 0.04 | 0.02 |  |  | 32 | 0.05 | 0.04 | 0.04 | 0.04 |  |  | 19 | 0.06 | 0.03 | 0.05 | 0.02 |  |  | 17 | 0.08 | 0.08 | 0.06 | 0.05 |  |  |

ASYM, asymmetry; DLS, double limb support; DLSV, double limb support variability; DT, dual task; IQR, interquartile range; M, mean; m/s, meter per seconds; n, sample size; s, seconds; SD, standard deviation; ST, single task; STV, step time variability; T1, time of measurement at admission; T2 time of measurement before discharge; W, test statistic of Wilcoxon signed-ranks test for dependent samples to calculated differences between T1 and T2 for each walking parameter, p≤0.05*, significant on level of significance α≤0.05.
